# Preventive induction of labor for non-urgent indications at term and maternal and neonatal outcomes

**DOI:** 10.1186/s12978-016-0165-5

**Published:** 2016-04-21

**Authors:** Lin Zhang, Hao Zhang, Jun Zhang, Jin Wen Zhang, Jiang Feng Ye, D. Ware Branch

**Affiliations:** Xinhua Hospital, Shanghai Jiao Tong University School of Medicine, 1665 Kongjiang Road, Shanghai, 200092 China; Intermountain HealthCare and University of Utah, Salt Lake City, UT USA

**Keywords:** Preventive induction of labor, Propensity score model, Cesarean delivery, Maternal outcomes, Neonatal outcomes

## Abstract

**Background:**

Induction of labor (IOL) is a common practice in many parts of the world. However, the benefits and risks of preventive IOL for the mother and baby have yet to be critically assessed. This study is to investigate the effects of preventive IOL for non-urgent indications at term on maternal and neonatal outcomes.

**Methods:**

In this study, we applied a propensity score model to mimic a randomized clinical trial. Maternal and neonatal outcomes were compared between women with preventive IOL at 37–39 weeks of gestation and women with ongoing pregnancy (expectant management). The subjects were from the Consortium on Safe Labor, a study of over 200,000 births from 19 hospitals across the US from 2002 to 2008.

**Results:**

Both nulliparous and multiparous women induced preventively for non-urgent indications at 37–38 weeks’ gestation had lower rates of cesarean delivery compared to those delivered at later gestational weeks. However, preventive IOL was associated with increased risks of adverse neonatal outcomes (adjusted odds ratio [aOR] = 1.68, 95 % confidence interval [CI], 0.97–2.92 for nulliparas; aOR = 2.22, 1.32–3.74 for multiparas) and admission to NICU (aOR = 1.48, 0.99–2.20 for nulliparas; aOR = 2.08, 1.47–2.96 for multiparas) at 37 weeks’ gestation. A longer maternal hospital stay was found among all women with preventive IOL.

**Conclusions:**

Preventive IOL for non-urgent indications may be associated with a decreased risk of cesarean delivery at early term but increased risks of adverse neonatal outcomes at 37 weeks. It also results in a longer hospital stay than expectant management.

## Background

Induction of labor is a common practice in many parts of the world [1]. More recently, “preventive (or proactive) induction” for women who have certain risk factors or non-urgent conditions for potentially unfavorable perinatal outcomes has been advocated [2, 3]. A Cochrane review [4] showed that planned early delivery versus expectant management for a suspected compromised fetus at term did not result in any differences in major outcomes of perinatal mortality, significant neonatal or maternal morbidity or neurodevelopmental disability. Nonetheless, the authors cautioned that the review was based on only one large trial and two smaller trials assessing fetuses with IUGR or oligohydramnios. Generalizability of the findings was limited. Thus, adequately-powered, randomized controlled trials are still needed to confirm these findings. Unfortunately, such trials are costly but previous large observational studies often suffer from important deficiencies in study design [5].

In a prospective, randomized trial, a woman would receive either labor induction at certain gestational week or expectant management to let the pregnancy continue in the absence of pre-defined indications for delivery. However, previous retrospective studies have compared induced with spontaneous labor at the same gestational week. Critics of the retrospective studies point out that as the pregnancy continues, certain risks accumulate. Randomized trials indicate, for instance, that induction at 41 weeks or later reduces the risk of cesarean delivery [6]. On the other hand, induction before 39 weeks may be associated with suboptimal neonatal outcomes, and failed induction is not uncommon particularly in nulliparas.

While awaiting large randomized trials to provide a balanced, definitive answer, there may be opportunities to use retrospective data to examine this issue with an appropriate methodology. Despite that preventive induction of labor is commonplace in some institutions, many women with the same non-urgent conditions are managed expectantly. We reasoned that retrospectively obtained data could be utilized to select pregnant women who are clinically comparable, but among whom one group received induction at a given gestational week (37–39 weeks) and the other continued the pregnancy until later gestational weeks. These two groups could then be used to compare the perinatal outcomes. Thus, using data from the Consortium on Safe Labor, a large, multicenter retrospective study, we employed a propensity score analysis and intention-to-treat principle to mimic a randomized clinical trial. Our goal is to provide further evidence regarding whether preventive induction of labor at 37–39 weeks of gestation improves or adversely affects maternal and neonatal outcomes.

## Methods

### Conceptual framework of the analysis

**Fig. 1:**
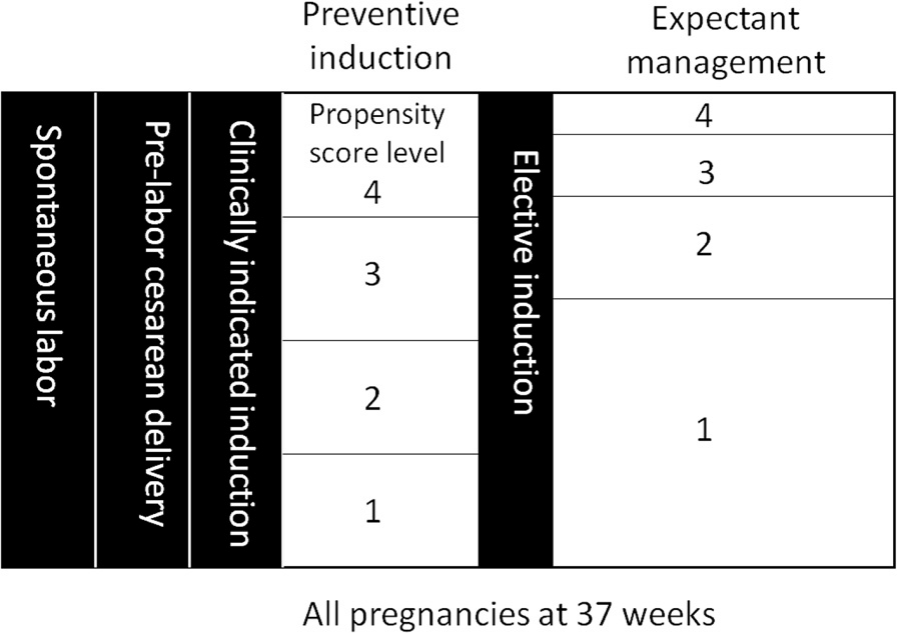
A conceptual framework in selecting compatible subjects for comparison. The intervention group (preventive induction) and expectant management (ongoing pregnancies as control group) at a particular gestational age (e.g., 37 weeks) were further divided based on the propensity score for preventive induction. Propensity score levels are divided based on the propensity scores in the preventive induction group (<25th, 25th–49th, 50th–74th, and ≥ 75th percentiles). These cut-off points were then applied to the expectant management group. 1 = least likely to have preventive induction; 4 = most likely to have preventive induction. Comparisons were made between the intervention and control groups at the corresponding propensity score level

The objective of this study is to compare women who had preventive induction of labor at a certain gestational age with women who had similar characteristics and conditions but were managed expectantly, i.e., their pregnancies continued beyond that gestational age. Figure 1 illustrates the conceptual framework. For example, at 37 weeks of gestation, most women would continue their pregnancies beyond 37 weeks. However, some would be delivered because of spontaneous onset of labor, prelabor cesarean (elective or repeat), or well-accepted clinical indications. Because such subjects would be excluded from a randomized controlled trial to test whether “preventive” labor induction at 37 weeks of gestation improves perinatal outcomes, we also excluded them from our analysis. Among the remaining cohort of women at 37 weeks gestation, those undergoing “preventive” induction of labor for non-urgent indications would be the intervention group and women continuing pregnancy would be potential controls. Further selection criteria are applied for final inclusion in the analysis (see below). The same approach would be used to construct 38-week and 39-week cohorts. These consecutive cohorts will help to answer when is the best time to perform preventive induction, if appropriate.

In our dataset, most women in the induction group had non-urgent conditions of varying degrees of severity, while the majority of expectant management group did not. To select women who were likely to be qualified candidates for preventive induction, we used propensity score analysis to assign each woman a score based on their characteristics and conditions [7, 8]. A high score represents a high likelihood of preventive induction. In the intervention group, most women had a high score while the majority of expectant management group had a low score. We then divided the intervention group into four subgroups based on quartiles of the propensity score in that group. We applied the same cut-off points to the expectant management group (Fig. 1). Stratified analyses based on the level of the propensity score were conducted to compare maternal and neonatal outcomes between the intervention and expectant management groups at the corresponding level.

### Study population

We used data from the Consortium on Safe Labor. Detailed description of the study population is provided elsewhere [9]. Briefly, the Consortium included 12 clinical centers (with 19 hospitals) across 9 American Congress of Obstetricians and Gynecologists (ACOG) districts. The study included 228,562 deliveries with 233,730 newborns in 2002–2008; 87 % of the births occurred in 2005–2007. All births at ≥23 weeks in these institutions were included. Participating institutions extracted detailed information from their electronic medical records. Obstetric information was linked to the newborn and neonatal intensive care unit (NICU) records. Information on hospital and physician characteristics was collected from surveys of the local investigators. Maternal and newborn discharge summaries (in ICD-9 codes) were linked to each delivery. Validation studies indicated that the electronic medical records are accurate representation of the medical charts in our study. To avoid potential correlation between pregnancies of the same individual, we selected only an individual’s first pregnancy in the study.

The Consortium on Safe Labor was approved by the institutional review boards of the National Institutes of Health, U.S.A. (IRB #2007-0656, date of approval 9/22/2008) and all participating institutions. An individual informed consent was not obtained because medical records were extracted without personal identification. Analyzing the de-identified data for scientific publication was exempted from IRB reviews.

### Preventive Induction Group

For the preventive induction group, we first selected subjects who had a singleton gestation, vertex presentation, no uterine scar or fetal congenital anomalies, and were delivered between 37 weeks +0 day and 39 weeks +6 days. We excluded women who had spontaneous onset of labor, premature rupture of the fetal membranes, placenta previa, pre-labor cesarean section, clinically-indicated induction or elective induction without any obstetric indication, or antepartum complications (fetal distress, chorioamnionitis, antepartum fetal death, thrombosis, cerclage and active herpes).

In the current analysis, preventive induction is defined as labor induction for any of the following reasons: hypertensive disorders (gestational hypertension, chronic hypertension, unspecified hypertension excluding preeclampsia, eclampsia and superimposed preeclampsia); maternal medical problems (e.g., preexisting or gestational diabetes, history of heart disease or renal disease); isolated oligohydramnios or small-for-gestational-age; polyhydramnios; history of maternal or fetal conditions in prior pregnancy; suspected macrosomia; vaginal bleeding in 3rd trimester (but not immediately before labor).

### Expectant management group

For the expectant management group, we selected subjects who had a singleton gestation, vertex presentation, no uterine scar or fetal congenital anomalies, and were delivered at 38 weeks +0 day or later. We excluded women who had placenta previa or cerclage. Note that the expectant management group could be delivered by any method for any reason after the “enrollment window”.

We then divided the induction group into five subsets according to the time when preventive induction was performed: 37^+0^–37^+6^, 38^+0^–38^+3^, 38^+4^–38^+6^, 39^+0^–39^+3^, and 39^+4^–39^+6^ weeks. The corresponding expectant groups were all eligible women who were delivered at ≥ 38^+0^, ≥ 38^+4^, ≥ 39^+0^, ≥ 39^+4^ and ≥ 40^+0^ weeks, respectively (Table 1). Thus, we had five cohorts with different induction times.

**Table 1:** Distribution of study subjects (Number of subjects in each subgroup)

	Preventive induction				Expectant management			
Propensity score level*	1	2	3	4	1	2	3	4
Gestational age at delivery	37^+0^–37^+6^ week				≥38^+0^ week			
Nulliparas	128	129	128	129	50530	6785	3233	707
Multiparas	169	170	170	170	45524	11118	3191	809
Gestational age at delivery	38^+0^–38^+3^week				≥38^+4^week			
Nulliparas	175	176	175	176	39865	8098	3272	709
Multiparas	249	249	249	249	35869	9095	3253	896
Gestational age at delivery	38^+4^–38^+6^ week				≥39^+0^week			
Nulliparas	85	85	85	85	42454	3774	1486	254
Multiparas	96	97	97	97	36355	5728	2077	673
Gestational age at delivery	39^+0^–39^+3^week				≥39^+4^week			
Nulliparas	251	252	251	252	21715	7690	1918	619
Multiparas	416	417	416	417	17639	3257	2457	502
Gestational age at delivery	39^+4^–39^+6^week				≥40^+0^ week			
Nulliparas	52	53	52	53	21612	3178	1570	755
Multiparas	59	59	59	59	15437	3104	856	326

*Propensity score levels are divided based on the propensity scores in the preventive induction group (<25th, 25th–49th, 50th–74th, and ≥ 75th percentiles). These cut-off points were then applied to the expectant management group

### Variable classification

We included a wide range of predictors (from maternal demographic characteristics, underlying pregnancy complications to physician and hospital characteristics) in the propensity score model. A complete list of variables that are included in the model is provided in the Appendix 1. Our outcome measures include: cesarean delivery, maternal and neonatal complications, admission to neonatal intensive care unit, and duration of maternal hospital stay. Since severe maternal and neonatal complications were rare, we created maternal and neonatal composite indexes. The maternal composite index includes maternal intensive care unit admission, postpartum hemorrhage, postpartum blood transfusion, shoulder dystocia, endometritis, and 3rd or 4th degree perineal laceration. The neonatal composite index includes stillbirth, birth injury, Apgar at 5 minutes < 4, neonatal sepsis, neonatal pneumonia, intracranial hemorrhage, asphyxia, hemorrhagic-ischemic encephalopathy, respiratory distress syndrome, neonatal seizure, periventricular-intraventricular hemorrhage, necrotizing enterocolitis, apnea, retinopathy of prematurity, neonatal anemia, patent ductus arteriosis, ventilation, blood transfusion, neonatal death, and pneumonia due to aspiration or infection.

### Data analysis

We first performed stratified analyses, comparing the incidence of adverse outcomes between the induction and expectant groups separated by the level of propensity score, gestational age and parity. We then used a log binomial model to produce relative risks of adverse outcomes after adjusting for the propensity score [7]. A multiple linear regression was used to examine the adjusted difference in duration of maternal hospital stay. All analyses were performed using SAS 9.3.

## Results

Table 1 presents the number of subjects under each category. In the preventive induction group, the total number of subjects was equally divided based on the quartile of the propensity score in that group. When the same cut-off points of the propensity score were applied to the expectant group, the vast majority of the women had a low level of propensity score.

Table 2 shows that in nulliparas, the rate of cesarean delivery increased with increasing propensity score. However, there was no difference in the composite maternal or neonatal adverse outcomes overall, though some sporadic differences were statistically significant. Similar findings were observed among multiparas (Table 3). These analyses indicate that the differences (or similarity) in the incidence of adverse outcomes between the induction and expectant groups did not vary substantially by the level of propensity score and the gestational age at induction. Therefore, we combined levels 3 and 4 of propensity score to increase the statistical power.

**Table 2:** Preventive induction vs. expectant management in relation to maternal and perinatal outcomes in nulliparas

Gestational	Propensity	Cesarean delivery			Maternal outcomes			Neonatal outcomes			NICU admission		
		Induction	Expectant		Induction	Expectant		Induction	Expectant		Induction	Expectant	
Age (week)	Score level												
		(%)	(%)	P	(%)	(%)	P	(%)	(%)	P	(%)	(%)	P
37^+0^–37^+6^	1	25.0	24.0		4.7	9.6		1.6	3.8		8.6	6.7	
	2	31.0	29.2		5.4	6.2		1.6	3.7		6.2	7.0	
	3	32.0	35.9		4.7	5.6		7.0	3.6	*	13.3	6.7	**
	4	31.0	43.1	*	4.7	8.1		3.9	2.8		7.0	6.4	
38^+0^–38^+3^	1	27.4	23.6		11.4	10.0		3.4	3.9		10.9	6.9	*
	2	26.1	36.6	**	8.5	6.9		3.4	3.3		9.1	6.9	
	3	24.0	25.6		12.0	5.1	***	2.9	3.7		4.6	5.5	
	4	37.5	43.3		6.3	6.2		2.3	3.0		5.1	6.8	
38^+4^–38^+6^	1	27.1	25.5		11.8	9.7		4.7	3.9		4.7	7.1	
	2	29.4	28.5		5.9	6.3		5.9	3.1		8.2	5.4	
	3	30.6	36.1		7.1	6.6		0.0	2.9		1.2	5.5	
	4	41.2	43.3		2.4	5.5		0.0	3.5		2.4	5.5	
39^+0^–39^+3^	1	34.3	27.9	*	8.8	8.5		2.4	4.2		4.0	7.6	*
	2	30.2	26.8		9.5	11.5		2.4	3.8		7.5	6.4	
	3	32.7	27.8		15.1	9.2	**	3.2	4.0		5.2	7.0	
	4	34.9	38.8		11.1	10.2		2.0	4.5		6.0	7.6	
39^+4^–39^+6^	1	17.3	27.9		15.4	10.3		3.8	4.4		5.8	7.9	
	2	39.6	31.8		11.3	5.8		0.0	3.5		1.9	4.8	
	3	38.5	28.3		5.8	7.3		1.9	3.6		3.8	5.6	
	4	32.1	37.2		11.3	4.6	*	3.8	2.9		5.7	6.6	

*p < 0.05; **p < 0.01; ***p < 0.001

**Table 3:** Preventive induction vs. expectant management in relation to maternal and perinatal outcomes inmultiparas

Gestational	Propensity	Cesarean delivery			Maternal outcomes			Neonatal outcomes			NICU admission		
		Induction	Expectant		Induction	Expectant		Induction	Expectant		Induction	Expectant	
Age (week)	Score level												
		(%)	(%)	P	(%)	(%)	P	(%)	(%)	P	(%)	(%)	P
37^+0^–37^+6^	1	8.3	5.5		9.5	7.3		3.0	2.0		7.7	4.0	*
	2	4.1	7.6		7.6	4.4	*	3.5	2.0		10.0	4.0	***
	3	6.5	10.4		4.1	3.5		4.7	2.1	*	10.0	4.9	***
	4	4.7	15.5	***	4.7	3.5		5.9	2.8	*	11.8	5.6	**
38^+0^–38^+3^	1	4.8	5.6		5.2	6.7		2.8	1.9		7.6	3.7	**
	2	4.0	6.8		7.6	6.7		2.4	2.0		6.8	3.9	*
	3	6.0	8.8		6.4	5.4		1.6	1.8		2.4	4.4	
	4	10.0	14.7		3.6	5.1		2.0	3.2		6.8	6.1	
38^+4^–38^+6^	1	5.2	6.0		9.4	7.1		4.2	2.0		4.2	4.0	
	2	3.1	5.8		8.2	5.9		1.0	1.4		1.0	2.7	
	3	7.2	8.8		3.1	4.3		1.0	1.7		1.0	3.7	
	4	3.1	9.4	*	1.0	2.2		1.0	1.5		2.1	2.8	
39^+0^–39^+3^	1	7.6	7.2		3.9	4.3		2.0	1.9		5.1	3.9	
	2	5.6	6.7		6.8	7.7		2.2	1.8		4.9	4.2	
	3	4.6	3.5		12.0	13.3		2.2	1.9		4.9	3.6	
	4	4.6	6.2		14.7	10.8		3.7	1.2	*	5.9	3.0	*
39^+4^–39^+6^	1	3.4	7.5		8.5	5.4		1.7	2.0		1.7	4.1	
	2	1.7	4.9		10.2	7.9		1.7	1.5		3.4	2.9	
	3	3.4	6.1		3.4	4.3		3.4	1.8		3.4	3.6	
	4	3.4	8.9		3.4	4.0		1.7	0.9		3.4	3.7	

*p < 0.05; **p < 0.01; ***p < 0.001

Table 4 demonstrates that in both nulliparas and multiparas, preventive induction at 37 and 38 weeks was associated with a reduced risk of cesarean delivery comparing to expectant management. No difference was observed at 39 weeks. However, at 37 weeks preventive induction was associated with a two-fold increased risk of adverse neonatal outcomes and NICU admission in multiparas. The trend appeared similar in nulliparas but less significant. Women with preventive induction stayed in the hospital significantly longer than women with expectant management. Among women with a propensity score at levels 1 and 2, preventive induction was associated with an increased risk of NICU admission at 37 and 38 weeks, and had longer hospital stay. (Appendix 2).

**Table 4:** Adjusted relative risk of adverse outcomes by gestational age at intervention among women with a propensity score at levels 3 and 4

Adverse outcome	Gestational age at induction/delivery	Nulliparas RR (95 % CI)^a^	Multiparas RR (95 % CI)^a^
Cesarean delivery	37^+0^–37^+6^ vs. ≥ 38^+0^	0.80 (0.67–0.97)	0.42 (0.26–0.65)
	38^+0^–38^+6^ vs. ≥ 39^+0^	0.83 (0.73–0.95)	0.65 (0.49–0.87)
	39^+0^–39^+6^ vs. ≥ 40^+0^	0.95 (0.84–1.08)	0.96 (0.66–1.39)
Maternal adverse outcome	37^+0^–37^+6^ vs. ≥ 38^+0^	0.69 (0.39–1.23)	1.25 (0.73–2.14)
	38^+0^–38^+6^ vs. ≥ 39^+0^	1.09 (0.76–1.55)	0.93 (0.63–1.36)
	39^+0^–39^+6^ vs. ≥ 40^+0^	1.39 (1.07–1.81)	1.02 (0.82–1.26)
Neonatal adverse outcome	37^+0^–37^+6^ vs. ≥ 38^+0^	1.68 (0.97–2.92)	2.22 (1.32–3.74)
	38^+0^–38^+6^ vs. ≥ 39^+0^	0.57 (0.30–1.10)	0.89 (0.49–1.63)
	39^+0^–39^+6^ vs. ≥ 40^+0^	0.75 (0.44–1.26)	1.79 (1.08–2.98)
NICU admission	37^+0^–37^+6^ vs. ≥ 38^+0^	1.48 (0.99–2.20)	2.08 (1.47–2.96)
	38^+0^–38^+6^ vs. ≥ 39^+0^	0.52 (0.32–0.84)	0.89 (0.60–1.34)
	39^+0^–39^+6^ vs. ≥ 40^+0^	0.87 (0.61–1.24)	1.47 (1.02–2.12)
		Mean difference (95 % CI, days)^b^	Mean difference (95 % CI, days)^b^
Maternal hospital stay	37^+0^–37^+6^ vs. ≥ 38^+0^	1.07 (1.02–1.12)	1.13 (1.09–1.18)
	38^+0^–38^+6^ vs. ≥ 39^+0^	1.04 (1.01–1.08)	1.06 (1.03–1.10)
	39^+0^–39^+6^ vs. ≥ 40^+0^	1.07 (1.03–1.10)	1.11 (1.08–1.14)

^a^log binomial model adjusting for propensity score. ^b^least squares mean difference adjusting for propensity score

## Discussion

Our study was a multicenter, retrospective study. There was no standard protocol for labor induction. Our data reflected the current common practice of labor induction and cesarean delivery in the U.S. [9]Therefore, the current study is more like an effectiveness trial (close to reality) than an efficacy trial (more selective and strictly controlled). We found that the major reduction in cesarean rate was concentrated at early term (37–38 weeks). However, unintended consequences with regard to unfavorable newborn outcomes were also observed at 37 weeks. The longer hospital stay for induced labor is another factor to be taken into account. It remains to be confirmed that a reduction in cesarean rate in this group can compensate for the increased risk of adverse neonatal outcomes and longer hospital stay.

Is the effectiveness of preventive induction complication-specific? The HYPITAT trial found that women with gestational hypertension or mild preeclampsia induced at 37–41 weeks’ gestation had a lower rate of cesarean section than those received expectant monitoring [10]. A historical cohort study suggested that more proactive post-term induction was associated with improved perinatal outcomes [11]. On the other hand, for women with uncomplicated insulin-requiring diabetes, no significant difference in cesarean rate was found [12]. Thus, it is possible that the benefit of preventive induction may vary by type of complications. Unfortunately, due to the limited number of subjects in our study, stratified analyses separated by each complication were not possible.

Our finding that preventive induction at 37 weeks may increase the risk of adverse neonatal outcomes is consistent with the results from recent HYPITAT-II trial [13]. The latter study randomized 754 women to either planned delivery at 34–36 weeks or expectant monitoring. It found that while adverse maternal outcomes occurred in 0.9 % and 2.8 % in the two groups, respectively, (RR = 0.30, 95 % CI 0.08–1.08), the incidence of neonatal respiratory distress syndrome in the intervention group more than doubled at late preterm.

It should also be noted that our study tried to mimic a randomized clinical trial using propensity score stratification and adjustment. This method of improving comparability has become more and more popular in biomedical research [8]. Further, as we had included a wide range of variables into this model, most treatment-selection bias may have been eliminated [14]. In addition, our study was able to separate by gestational week and pinpoint where the benefits and risks were.

The limitations of our study are also worth noting. Due to the nature of the observational study, non-documented factors that can influence the likelihood of preventive induction of labor may not have been included in the model. For instance, the status of the cervix might have played a role in deciding whether to perform preventive induction at early term or wait for spontaneous onset of labor. If this is true, we may expect to see that women who had preventive induction at early term had a decreased risk of cesarean delivery than the corresponding expectantly managed group due to more favorable cervical status. Unfortunately, we don’t have information on cervical ripeness at the time when the decision of preventive induction was made. Likewise, one may suspect that the increased risk of maternal and neonatal adverse outcomes might have been due to worse conditions leading to preventive induction, i.e., maternal and neonatal adverse outcomes were the cause rather than the consequences. However, variations in the incidence of adverse maternal and neonatal outcomes by the propensity score were small, suggesting that the potential bias introduced by the cervical ripeness and maternal and fetal conditions may not be substantial.

Another limitation of this study is its generalizability. To have enough statistical power, we combined women with various non-urgent medical conditions before stratifying them by parity, gestational age and propensity score. Women with the same score level had a comparable probability of preventive induction according to the current standard of care, but their obstetrical risks might be somewhat different because of different types of medical conditions. And our study was not powerful enough to analyze or discuss these factors individually. In summary, we found that preventive IOL for non-urgent indications may be associated with a decreased risk of cesarean delivery at early term but increased risks of adverse neonatal outcomes at 37 weeks. It also results in a longer hospital stay than expectant management. In order to develop a complete guidance on making clinical decision, large randomized controlled trials for specific obstetrical conditions such as gestational hypertension or diabetes are still warranted.

## Conclusions

Our study suggests that preventive induction for non-urgent medical and obstetrical conditions at early term (37–38 weeks of gestation) may be associated with a reduced risk of cesarean delivery. On the other hand, preventive induction at 37 weeks is associated with an increased risk of adverse neonatal outcomes. Women with induction stayed hospital longer than expectantly managed women.

## Appendix 1

Variables that were included in the propensity score model:

### Maternal demographic characteristics

Maternal age, race, education level, height, weight at admission to birth, marital status, insurance status;

### Medical history

History of macrosomia, preterm and still births, preexisting diabetes, chronic hypertension, heart, renal, gastrointestinal, thyroid diseases, depression, seizure, use of assisted reproductive technology;

### Antepartum complications/conditions

Smoking, alcohol drinking, drug abuse during pregnancy, hospitalization, sexually transmitted diseases, Group B streptococcus, urinary tract infection, 3rd trimester vaginal bleeding, anemia, asthma, hyper- or hypothyroidism, gestational hypertension, chronic hypertension, unspecified hypertension, gestational diabetes, threatened preterm birth, incompetent cervix, suspected small-for-gestational-age, suspected large-for-gestational-age, oligohydramnions, polyhydramnios, placental abruption;

### Hospital and delivering doctor’s characteristics

Sex and age of the physician, year of graduation, type of physician practice, type of doctor’s malpractice insurance, hospital type, hospital level, type of hospital insurance, maternal-fetal medicine staff, hospitalist staffing, use of midwife, neonatologist at delivery, NICU level, use of epidural nurse, elective induction allowed, elective cesarean delivery.

## Appendix 2

**Table 5:** Adjusted relative risk of adverse outcomes by gestational age at intervention among women with a propensity score at levels 1 and 2

Adverse outcome	Gestational age at induction/delivery	Nulliparas RR (95 % CI)^a^	Multiparas RR (95 % CI)^a^
Cesarean delivery	37^+0^–37^+6^ vs. ≥ 38^+0^	0.95 (0.78–1.15)	0.86 (0.57–1.30)
	38^+0^–38^+6^ vs. ≥ 39^+0^	0.92 (0.80–1.07)	0.67 (0.47–0.96)
	39^+0^–39^+6^ vs. ≥ 40^+0^	1.14 (1.01–1.29)	0.89 (0.69–1.16)
Maternal adverse outcome	37^+0^–37^+6^ vs. ≥ 38^+0^	0.79 (0.47–1.34)	1.66 (1.17–2.35)
	38^+0^–38^+6^ vs. ≥ 39^+0^	1.41 (1.09–1.81)	1.01 (0.77–1.33)
	39^+0^–39^+6^ vs. ≥ 40^+0^	1.07 (0.84–1.35)	0.86 (0.65–1.13)
Neonatal adverse outcome	37^+0^–37^+6^ vs. ≥ 38^+0^	0.41 (0.16–1.09)	1.69 (0.94–3.05)
	38^+0^–38^+6^ vs. ≥ 39^+0^	1.10 (0.71–1.70)	1.32 (0.81–2.17)
	39^+0^–39^+6^ vs. ≥ 40^+0^	0.53 (0.31–0.91)	1.01 (0.62–1.64)
NICU admission	37^+0^–37^+6^ vs. ≥ 38^+0^	1.08 (0.77–1.67)	2.27 (1.61–3.21)
	38^+0^–38^+6^ vs. ≥ 39^+0^	1.50 (1.14–1.98)	1.61 (1.17–2.19)
	39^+0^–39^+6^ vs. ≥ 40^+0^	0.74 (0.51–1.05)	1.27 (0.93–1.73)
		Mean difference (95 % CI, days)^b^	Mean difference (95 % CI, days)^b^
Maternal hospital stay	37^+0^–37^+6^ vs. ≥ 38^+0^	1.12 (1.07–1.18)	1.15 (1.10–1.21)
	38^+0^–38^+6^ vs. ≥ 39^+0^	1.08 (1.04–1.12)	1.10 (1.06–1.14)
	39^+0^–39^+6^ vs. ≥ 40^+0^	1.17 (1.13–1.20)	1.12 (1.08–1.16)

^a^log binomial model adjusting for propensity score. ^b^least squares mean difference adjusting for propensity score
